# Title: Is bariatric surgery an option for obesity in autism spectrum disorder?: A case report

**DOI:** 10.1192/j.eurpsy.2023.634

**Published:** 2023-07-19

**Authors:** Z. I. Erbasan, B. N. Özbaran, D. R. Gökşen Şimşek, H. G. Balkı

**Affiliations:** ^1^child and adolescent psychiatry; ^2^Pediatric Endocrinology, ege university faculty of medicine, izmir, Türkiye

## Abstract

**Introduction:**

Autism spectrum disorder (ASD) is a neurodevelopmental disorder characterized by early onset difficulties in social communication, restricted repetitive behaviors and interests, and sensory sensitivities/differences (1). It has been determined that 90% of children with ASD have nutritional problems (2). There are many factors affecting nutrition in children with ASD, such as gastrointestinal problems, food allergies, metabolic anomalies, drug side effects such as increased appetite, problematic eating behavior, sensory processing difficulties, and family factors (3).

**Objectives:**

In this case report; we aimed to represent multidisciplinary medical and psychiatric treatment of a 16 years old adolescent with autism spectrum disorder and obesity who was consulted for bariatric surgery. It is thought that this case will be useful for clinicians as an example of a multidisciplinary approach in the management of obesity, primarily with non-surgical, psychiatric approaches and therapeutic environment.

**Methods:**

In our patient, it was decided to prefer non-surgical approaches primarily, considering the diagnosis of ASD, MID, and ADHD, difficulties in impulse control, the concern about the continuation of the poor lifestyle habits after bariatric surgery, and the difficulties that the family may experience in providing adequate postoperative care. Patient received Metformin 2000 mg/day for insulin resistance, Amlodipine 10 mg/day, Enalapril 5 mg/day, Perindopril 5 mg/day and Indapamide 1.25 mg/day combination for hypertension, Haloperidol 0.6 mg/day in case of impulse control problems and aggression, Topiramate 50 mg/day to take advantage of its appetite suppressing effect and Methylphenidate 10 mg/day for attention deficit hyperactivity disorder (ADHD) during hospitalization in the endocrinology clinic and the same time diet and exercise programs were applied. When his rate of weight loss decreased around 4th week, Exenatide 10 mcg/day was added to his treatment.

**Results:**

It was observed that the patient lost 15 kilograms at the end of 3 weeks, and his BMI decreased from 60.9 to 56.1 (BMI SDS: +4,18) and the total TG level decreased from 195 mg / dl to 154 mg / dl.

**Conclusions:**

Obesity, which is an important public health problem, is also becoming a serious problem in individuals with ASD. There is no standard treatment approach for the coexistence of ASD and obesity. Whether psychopathologies constitute a definite contraindication for bariatric surgery is a controversial issue. In our patient, it was decided to prefer non-surgical approaches primarily. It can be concluded that; In children and adolescents with autism and intellectual disability, effective weight loss can be achieved without bariatric surgery with medical and psychiatric approaches.

**Disclosure of Interest:**

None Declared

